# Supplementary material for: Identification of risk factors of Long COVID and predictive modeling in the RECOVER EHR cohorts
Source: Commun Med (Lond). 2024 Jul 11;4:130. doi: 10.1038/s43856-024-00549-0 (PMC11239808; doi:10.1038/s43856-024-00549-0)
Supplement: Supplementary file 2 — Supplementary Information [file 43856_2024_549_MOESM2_ESM.pdf]

Supplementary Information

We acknowledge the following RECOVER-EHR Consortium Members: PCORnet Core Contributors-Louisiana Public Health Institute, Tom Carton, PI, Anna Legrand, Elizabeth Nauman; PCORnet Core Contributors-Weill Cornell Medicine, Rainu Kaushal, PI, Sajjad Abedian, Dominique Brown, Christopher Cameron, Thomas Campion, Andrea Cohen, Marietou Dione, Rosie Ferris, Wilson Jacobs, Michael Koropsak, Alex LaMar, Colby V. Lewis, Dmitry Morozyuk, Peter Morrisey, Duncan Orlander, Jyotishman Pathak, Mahfuza Sabiha, Edward J. Schenck, Stephenson Strobel, Zoe Verzani, Fei Wang, Mark Weiner, Zhenxing Xu, Chengxi Zang, Yongkang Zhang; PCORnet Data Contributors, Albert Einstein College of Medicine Parsa Mirhaji, PI | Columbia University Soumitra Sengupta, PI, | Emory University Rishi Kamaleswaran, PI | Icahn School of Medicine at Mount Sinai Carol R. Horowitz, PI | New York University Langone Health Saul Blecker, PI | University of Florida Mei Liu, PI, | University of Miami Nick Tsinoremas, PI | University of South Florida | Nicklaus Children's Hospital, Sandy Gonzalez, PI | Weill Cornell Medicine Rainu Kaushal, PI

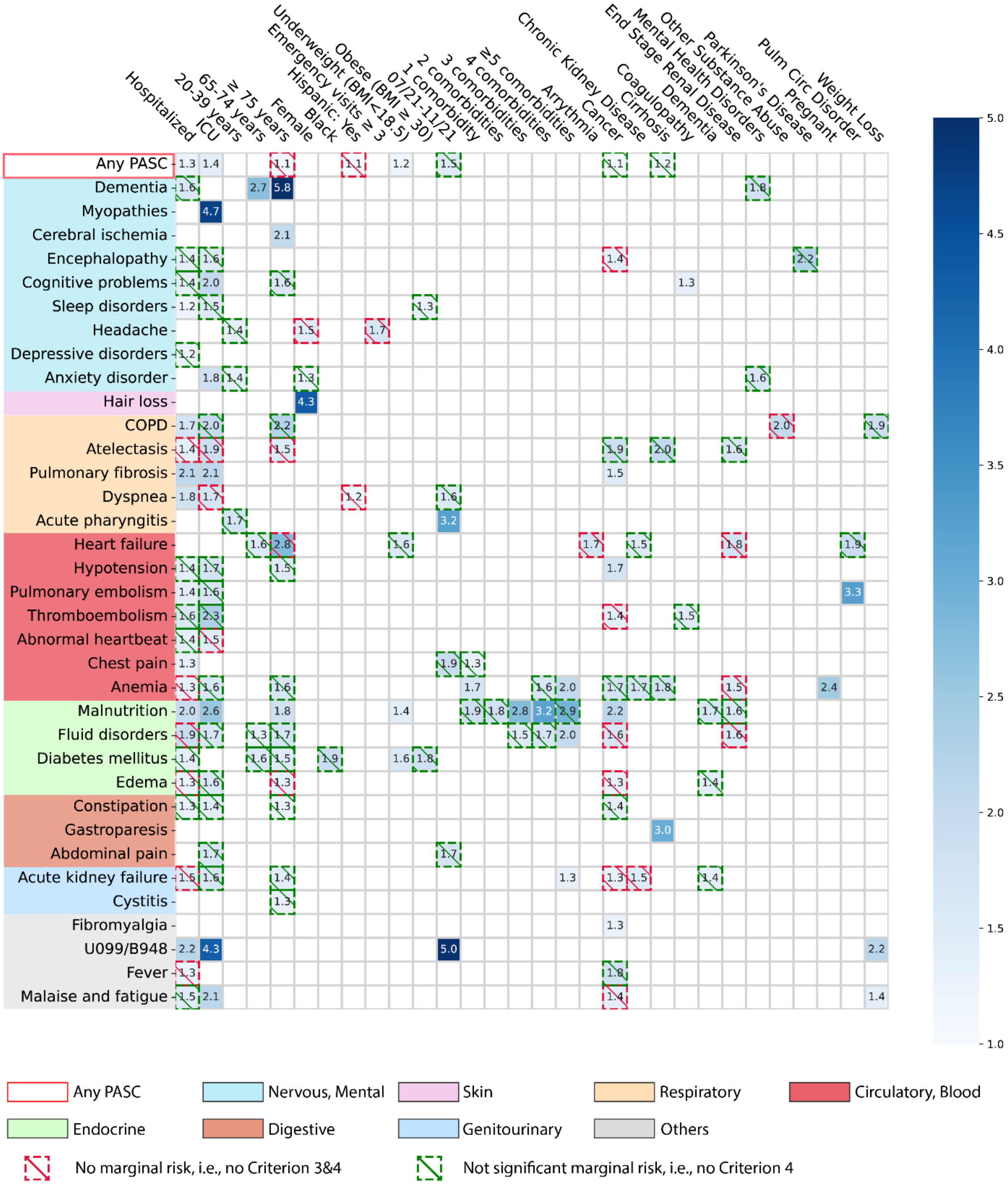
Hospitalized
20-39 years
ICU
65-74 years
≥ 75 years
Female
Black
Hispanic: Yes
Emergency visits ≥ 3
Underweight (BMI <18.5)
Obese (BMI ≥ 30)
07/21-11/21
1 comorbidity
2 comorbidities
3 comorbidities
4 comorbidities
≥5 comorbidities
Arrythmia
Cancer
Chronic Kidney Disease
Cirrhosis
Coagulopathy
Dementia
End Stage Renal Disease
Mental Health Disorders
Other Substance Abuse
Parkinson's Disease
Pregnant
Pulm Circ Disorder
Weight Loss
Any PASC
Dementia
Myopathies
Cerebral ischemia
Encephalopathy
Cognitive problems
Sleep disorders
Headache
Depressive disorders
Anxiety disorder
Hair loss
COPD
Atelectasis
Pulmonary fibrosis
Dyspnea
Acute pharyngitis
Heart failure
Hypotension
Pulmonary embolism
Thromboembolism
Abnormal heartbeat
Chest pain
Anemia
Malnutrition
Fluid disorders
Diabetes mellitus
Edema
Constipation
Gastroparesis
Abdominal pain
Acute kidney failure
Cystitis
Fibromyalgia
U099/B948
Fever
Malaise and fatigue
5.0
4.5
4.0
3.5
3.0
2.5
2.0
1.5
1.0
Any PASC
Nervous, Mental
Skin
Respiratory
Circulatory, Blood
Endocrine
Digestive
Genitourinary
Others
No marginal risk, i.e., no Criterion 3&4
Not significant marginal risk, i.e., no Criterion 4

Supplementary Fig. 1. Sensitivity analyses of associations ruled out by screening criteria 3 or 4, the INSIGHT cohort, March 2020 to November 2021. The associations ruled out by Criterion 3, namely marginal risk than the control group, were highlighted in red squares, and the associations ruled out by Criterion 4, namely the marginal risk than the control group should be significant, were highlighted in green squares. See details of screen criteria in the Method-association analysis section. The adjusted hazard ratios of different factors were reported. The colors represent different risk levels. Any PASC represents having at least one of the conditions below. The color panels represent different organ systems, including (from top to bottom): the nervous system or mental disorders, skin, respiratory system, circulatory system, endocrine and metabolic, digestive system, genitourinary system, and other signs. ICD-10 codes B948 (sequelae of other specified infectious and parasitic diseases) and U099 (post-COVID-19 condition, unspecified) were used to capture general PASC diagnoses.

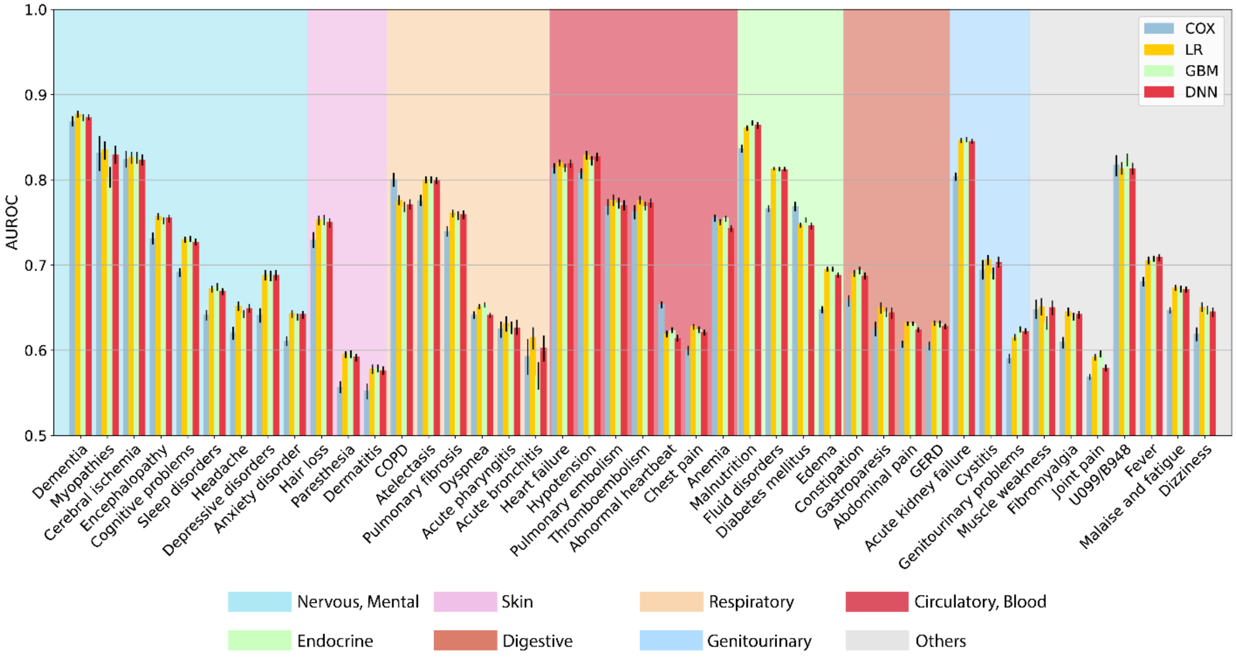

Supplementary Fig. 2. Predictive performance of incident post-acute sequelae of SARS-CoV-2 infection (PASC) by different machine learning models, based on the pre-defined baseline covariates and the severity of acute infection, INSIGHT, March 2020 to November 2021. The pre-defined baseline covariates were the same ones used in the risk factor analysis. The COX model was measured by C-index. Regularized logistic regression (LR), gradient boosting machine (GBM), and deep forward neural network (DNN) were measured by the area under the receiver operating characteristic curve (AUROC). The color panels represent different organ systems, including (from top to bottom): the nervous system or mental disorders, skin, respiratory system, circulatory system, endocrine and metabolic, digestive system, genitourinary system, and other signs. The error bars are 95% confidence interval estimated by 1000-times bootstrapping performance on the testing dataset in repeated cross-validation.

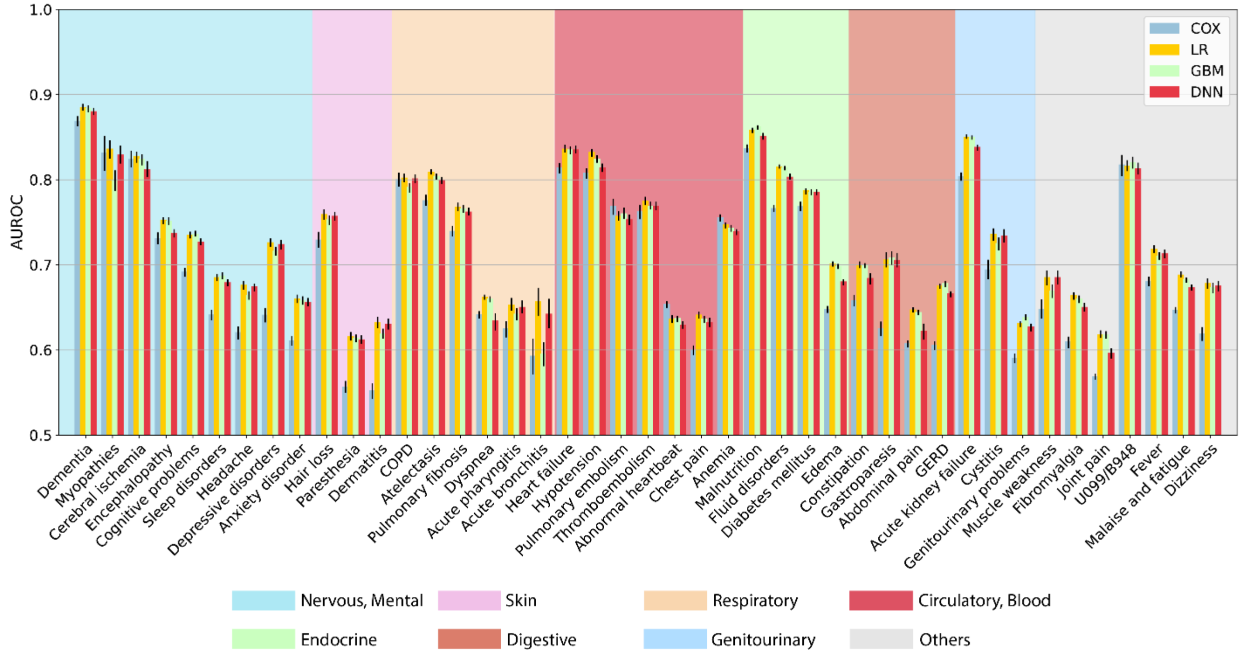

Supplementary Fig. 3. Predictive performance of incident post-acute sequelae of SARS-CoV-2 infection (PASC) based on building baseline covariates in a data-driven way, by different machine learning models, INSIGHT, March 2020 to November 2021. The data-driven baseline covariates were selected based on the first 3 digits of ICD-10 codes and prescribed medications at the ingredient level in a data-driven way. The COX model was measured by C-index. Regularized logistic regression (LR), gradient boosting machine (GBM), and deep forward neural network (DNN) were measured by the area under the receiver operating characteristic curve (AUROC). The color panels represent different organ systems, including (from top to bottom): the nervous system or mental disorders, skin, respiratory system, circulatory system, endocrine and metabolic, digestive system, genitourinary system, and other signs. The error bars are 95% confidence interval estimated by 1000-times bootstrapping performance on the testing dataset in repeated cross-validation.

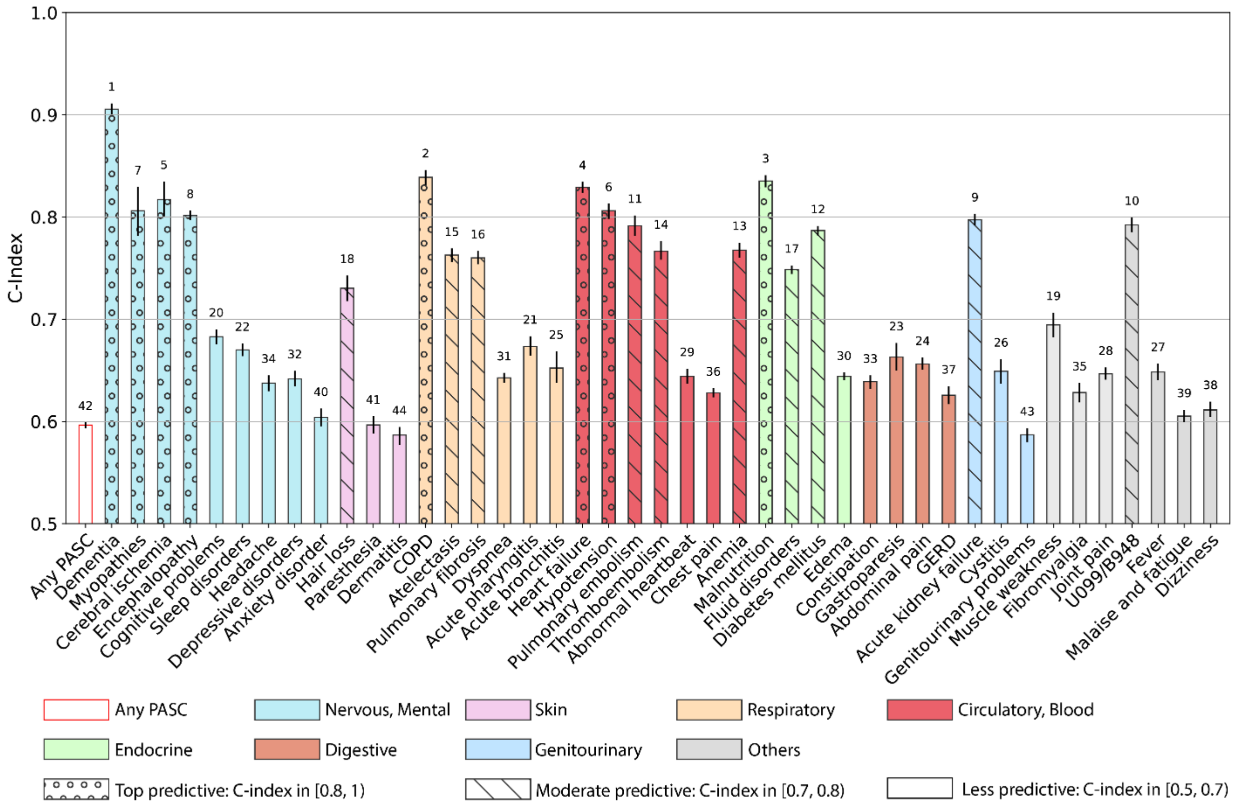

Supplementary Fig. 4. Prediction performance of incident potential PASC conditions from baseline characteristics and the severity in the acute phase with the OneFlorida+ cohort from March 2020 to November 2021. The C-index with a 95% confidence interval was reported. Any PASC represents having at least one of the conditions below. The bars in different colors were organized by their organ systems including (from left to right): the nervous system or mental disorders, skin, respiratory system, circulatory system, endocrine and metabolic, digestive system, genitourinary system, and other signs. The conditions with a C-index in [0.8, 1) were highlighted with "o" texture, and those with a C-index in [0.7, 0.8) were highlighted with "\" texture. The numbers at the top of the bars denote the rank of the predictability quantified by the C-index among all the bars with 1 representing the top predictive condition. The error bars are 95% confidence interval estimated by 1000-times bootstrapping performance on the testing dataset in repeated cross-validation.

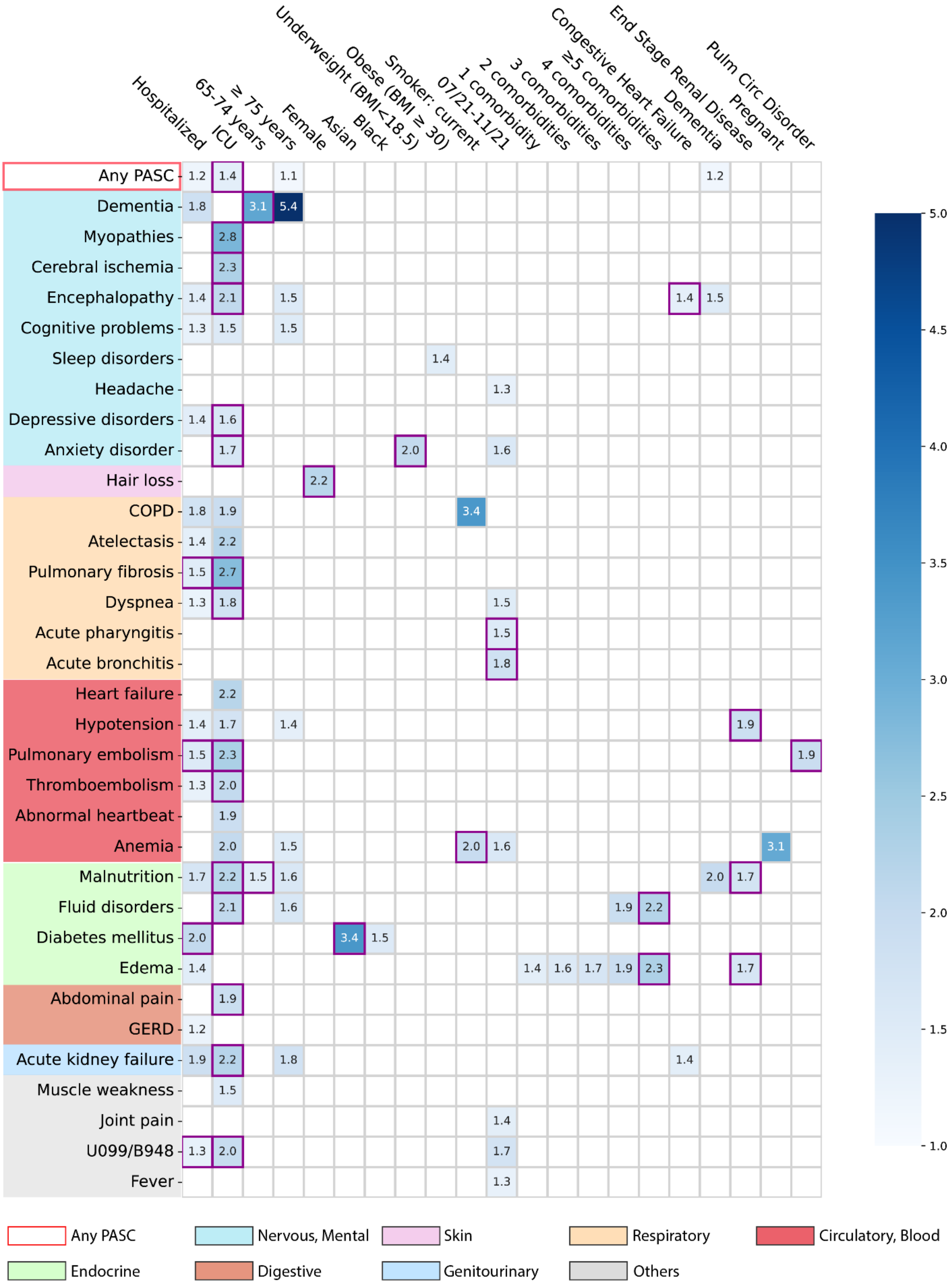
Hospitalized
ICU
65-74 years
≥ 75 years
Female
Asian
Black
Underweight (BMI<18.5)
Obese (BMI ≥ 30)
Smoker: current
07/21-11/21
1 comorbidity
2 comorbidities
3 comorbidities
4 comorbidities
≥5 comorbidities
Congestive Heart Failure
Dementia
End Stage Renal Disease
Pregnant
Pulm Circ Disorder
Any PASC
Dementia
Myopathies
Cerebral ischemia
Encephalopathy
Cognitive problems
Sleep disorders
Headache
Depressive disorders
Anxiety disorder
Hair loss
COPD
Atelectasis
Pulmonary fibrosis
Dyspnea
Acute pharyngitis
Acute bronchitis
Heart failure
Hypotension
Pulmonary embolism
Thromboembolism
Abnormal heartbeat
Anemia
Malnutrition
Fluid disorders
Diabetes mellitus
Edema
Abdominal pain
GERD
Acute kidney failure
Muscle weakness
Joint pain
U099/B948
Fever
5.0
4.5
4.0
3.5
3.0
2.5
2.0
1.5
1.0
Any PASC
Nervous, Mental
Skin
Respiratory
Circulatory, Blood
Endocrine
Digestive
Genitourinary
Others

Supplementary Fig. 5. Identified risk factors associated with incident PASC conditions from the OneFlorida+ cohort, March 2020 to November 2021. The adjusted hazard ratios of different factors were reported. The colors represent different risk levels. The associations whose marginal increased risks than non-infected control patients were also significant were highlighted in purple squares. Any PASC represents having at least one of the conditions below. The color panels represent different organ systems, including (from top to bottom): the nervous system or mental disorders, skin, respiratory system, circulatory system, endocrine and metabolic, digestive system, genitourinary system, and other signs. ICD-10 codes B948 (sequelae of other specified infectious and parasitic diseases) and U099 (post-COVID-19 condition, unspecified) were used to capture general PASC diagnoses.
