# Supplementary material for: Identification of risk factors of Long COVID and predictive modeling in the RECOVER EHR cohorts
Source: Commun Med (Lond). 2024 Jul 11;4:130. doi: 10.1038/s43856-024-00549-0 (PMC11239808; doi:10.1038/s43856-024-00549-0)
Supplement: Supplementary file 3 — Description of Additional Supplementary Files [file 43856_2024_549_MOESM3_ESM.pdf]

Description of Additional Supplementary Files

File name: Supplementary Data 1
Description: Diagnostic codes for adult PASC conditions

File name: Supplementary Data 2
Description: Sources data for main figures
