# Supplementary material for: Identification of risk factors of Long COVID and predictive modeling in the RECOVER EHR cohorts
Source: Commun Med (Lond). 2024 Jul 11;4:130. doi: 10.1038/s43856-024-00549-0 (PMC11239808; doi:10.1038/s43856-024-00549-0)
Supplement: Supplementary file 6 — Reporting Summary [file 43856_2024_549_MOESM6_ESM.pdf]

nature portfolio

Corresponding author(s): Fei Wang

Last updated by author(s): May 20, 2024

Reporting Summary

Nature Portfolio wishes to improve the reproducibility of the work that we publish. This form provides structure for consistency and transparency in reporting. For further information on Nature Portfolio policies, see our Editorial Policies and the Editorial Policy Checklist.

Statistics

For all statistical analyses, confirm that the following items are present in the figure legend, table legend, main text, or Methods section.

n/a	Confirmed	
☐	☒	The exact sample size (*n*) for each experimental group/condition, given as a discrete number and unit of measurement
☐	☒	A statement on whether measurements were taken from distinct samples or whether the same sample was measured repeatedly
☐	☒	The statistical test(s) used AND whether they are one- or two-sided<br>*Only common tests should be described solely by name; describe more complex techniques in the Methods section.*
☐	☒	A description of all covariates tested
☐	☒	A description of any assumptions or corrections, such as tests of normality and adjustment for multiple comparisons
☐	☒	A full description of the statistical parameters including central tendency (e.g. means) or other basic estimates (e.g. regression coefficient) AND variation (e.g. standard deviation) or associated estimates of uncertainty (e.g. confidence intervals)
☐	☒	For null hypothesis testing, the test statistic (e.g. *F*, *t*, *r*) with confidence intervals, effect sizes, degrees of freedom and *P* value noted<br>*Give P values as exact values whenever suitable.*
☒	☐	For Bayesian analysis, information on the choice of priors and Markov chain Monte Carlo settings
☒	☐	For hierarchical and complex designs, identification of the appropriate level for tests and full reporting of outcomes
☒	☐	Estimates of effect sizes (e.g. Cohen's *d*, Pearson's *r*), indicating how they were calculated

Our web collection on statistics for biologists contains articles on many of the points above.

Software and code

Policy information about availability of computer code

Data collection	No particular software was used for collecting the data.
Data analysis	For reproducibility, our codes are available at https://github.com/calvin-zcx/pasc_phenotype/prediction. We used Python 3.9, python package lifelines-0.2666 for survival analysis, and scikit-learn package 1.0.2 and LightGBM package 3.3.2 for machine learning models

For manuscripts utilizing custom algorithms or software that are central to the research but not yet described in published literature, software must be made available to editors and reviewers. We strongly encourage code deposition in a community repository (e.g. GitHub). See the Nature Portfolio guidelines for submitting code & software for further information.

Data

Policy information about availability of data

All manuscripts must include a data availability statement. This statement should provide the following information, where applicable:

- Accession codes, unique identifiers, or web links for publicly available datasets
- A description of any restrictions on data availability
- For clinical datasets or third party data, please ensure that the statement adheres to our policy

The INSIGHT data can be requested through https://insightcrn.org/. The OneFlorida+ data can be requested through https://onefloridaconsortium.org. Both the INSIGHT and the OneFlorida+ data are HIPAA-limited. Therefore, data use agreements must be established with the INSIGHT and OneFlorida+ networks. The relevant raw data for each figure are provided in the Supplementary Data 2-Source Data file.

Human research participants

Policy information about studies involving human research participants and Sex and Gender in Research.

Reporting on sex and gender	This is a retrospective secondary analysis of deidentified patient records from two large electronic health record (EHR) cohorts. Summary statistics on sex distributions within different cohorts were reported in Table 1. and extended data table 1
Population characteristics	We summarized the baseline characteristics of both the INSIGHT cohort and OneFlorida+ cohort in Table 1 from information that was available on patients in clinical data; demographic information was collected from patients when they registered for care within the healthcare systems. We observed significant differences between the two cohorts regarding age, gender, race, area deprivation index, and outbreak waves. The INSIGHT cohort contained SARS-CoV-2 infected patients mainly from the New York metropolitan area with the median area deprivation index (ADI, rankings from 1 to 100, with 1 and 100 indicating the lowest and highest level of disadvantage)17 15 (6-24) in the SARS-CoV-2 infected patient group, indicating fewer disadvantaged neighborhoods than the OneFlorida+ cohort whose median ADI was 58 (41-76). Indeed, the OneFlorida + cohort consisted of a mixture of urban, suburban, and rural populations in Florida and selected cities in Georgia and Alabama (see Methods). The median age of SARS-CoV-2 infected patients in the INSIGHT cohort was 55 (38-68), older than the OneFlorida+ cohort with a median age of 50 (34-64). Plus, more female SARS-CoV-2 infected patients were in the OneFlorida+ cohort (62.7%) than in the INSIGHT cohort (58.6%). The INSIGHT cohort also had a more diverse population with 34.7% white and 54.9% others (Asian and others including American Indian or Alaska Native, Native Hawaiian or other Pacific Islander, multiple races, etc.); the OneFlorida+ cohort had a majority of patients identifying as White race (51.0%). Additionally, there is a higher proportion of patients infected early in the pandemic in the INSIGHT cohort (31.8% of all infected patients were from March 2020 to June 2020) compared to the OneFlorida+ cohort (9.1% of cases were from March 2020 to June 2020). Different temporal patterns of new cases per month across two cohorts are illustrated in Extended Data Fig. 1. The two networks also differed in care settings connected to patient encounters and treatments utilized for infected patients (e.g., more inpatient visits and more prescriptions of corticosteroids in the OneFlorida+ cohort than in the INSIGHT cohort).
Recruitment	This is a retrospective secondary analysis of EHR data and no patient recruitment activities are involved.
Ethics oversight	The use of the INSIGHT data was approved by the Institutional Review Board (IRB) of Weill Cornell Medicine following protocol 21-10-95-380 with title “Adult PCORnet-PASC Response to the Proposed Revised Milestones for the PASC EHR/ ORWD Teams (RECOVER)”. The use of the OneFlorida+ data for this study was approved under the University of Florida IRB number IRB202001831. All EHRs used in this study were appropriately deidentified and thus no informed consent from patients was obtained.

Note that full information on the approval of the study protocol must also be provided in the manuscript.

Field-specific reporting

Please select the one below that is the best fit for your research. If you are not sure, read the appropriate sections before making your selection.

☒ Life sciences ☐ Behavioural & social sciences ☐ Ecological, evolutionary & environmental sciences

For a reference copy of the document with all sections, see nature.com/documents/nr-reporting-summary-flat.pdf

Life sciences study design

All studies must disclose on these points even when the disclosure is negative.

Sample size	We developed our primary results on the INSIGHT cohort and used the OneFlorida+ cohort as a validation cohort. Both cohorts were collected from patients who has at least one PCR/antigen test for SARS-CoV-2 infection from March 2020 to November 2021, and the inclusion-exclusion cascade was provided in Figure 1. The INSIGHT cohort included 35,275 adult patients with lab-confirmed SARS-CoV-2 infection and 326,126 non-infected control patients.
Data exclusions	For the both cohorts, adult patients (age ≥ 20) with at least one SARS-CoV-2 polymerase-chain-reaction (PCR) or antigen laboratory test (Supplemental Table 1) between March 01, 2020 and November 30, 2021 were selected. Then we chose the patients who had at least one positive test and had at least one potential PASC conditions in the follow-up (or post-acute infection) period defined as below. We further made sure those potential PASC conditions were new incidences in the follow-up period by excluding patients who had any of them in both baseline and follow-up periods. The overall inclusion-exclusion cascade was shown in Figure 1, and the relevant definitions are provided below.<br><br>Index date: the date of the first COVID-19 positive test.<br>Baseline period: from 3 years to one week prior to the index date.<br>Follow-up (post-acute infection) period: from 31 days after the index date to the day of documented death, last record in the database, 180 days after baseline, or the end of our observational window (Nov. 30, 2021), whichever came first.
Replication	The main analysis was conducted on the INSIGHT cohort and a replication analysis was done on the OneFlorida+ cohort.

Randomization	This is a retrospective analysis based on clustering and no randomization procedure was involved as no treatment effect was assessed.
Blinding	This is a retrospective analysis and thus blinding is not relevant.

Reporting for specific materials, systems and methods

We require information from authors about some types of materials, experimental systems and methods used in many studies. Here, indicate whether each material, system or method listed is relevant to your study. If you are not sure if a list item applies to your research, read the appropriate section before selecting a response.

Materials & experimental systems

n/a	Involved in the study
☒	☐ Antibodies
☒	☐ Eukaryotic cell lines
☒	☐ Palaeontology and archaeology
☒	☐ Animals and other organisms
☒	☐ Clinical data
☒	☐ Dual use research of concern

Methods

n/a	Involved in the study
☒	☐ ChIP-seq
☒	☐ Flow cytometry
☒	☐ MRI-based neuroimaging
